# A Supramolecular Polymerization Approach to the Growth of the Myofibril

**DOI:** 10.3389/fchem.2019.00487

**Published:** 2019-07-16

**Authors:** Alberto Ciferri, Alvin L. Crumbliss

**Affiliations:** Chemistry Department, Duke University, Durham, NC, United States

**Keywords:** supramolecular polymerization, sarcomers, myofibrils, actin, myosin, tinin, α-actinin

## Abstract

Extended linear structures self-assemble by the multi-stage-open-association mechanism of supramolecular polymerization (MSOA). Application of the model requires the identification of a repeating unit, the main-chain supramolecular bond, and the binding constant. The strength of the bond and the degree of polymerization become extremely large when multiple sites for non-covalent interactions occur. These expectations had been previously verified in the case of the neuronal axon, for which the above parameters were assessed from its known molecular structure. The more complex case of the myofibril is analyzed here. The specific interactions that connect neighboring sarcomers have been a matter of debate. Recent work has focused on the bond between titin and α-actinin localized at the terminal Z-zones of each sarcomer. Elaboration of literature data suggests that titin-α-actinin interactions do bridge neighboring sarcomers, promoting the polymerization of myofibrils that attain macroscopic dimensions consistently with the MSOA predictions. The rationale for the complex structuration of single sarcomers is discussed.

## Introduction

**Figure 1:**
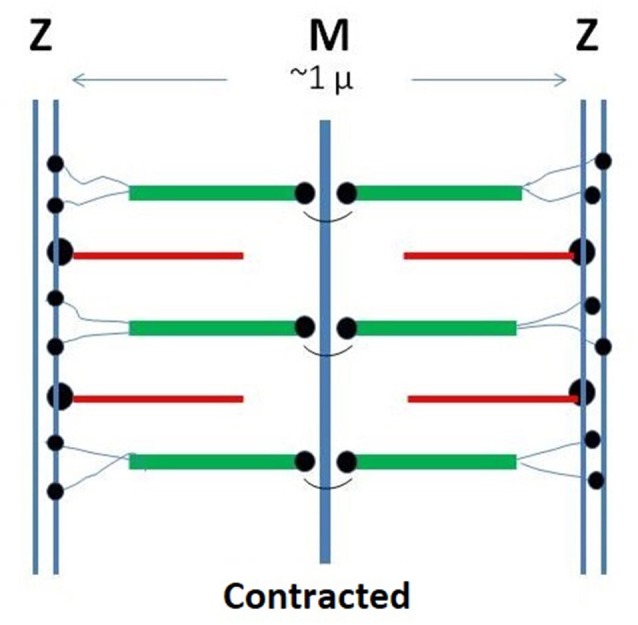
Schematic representation of the monomeric sarcomer. Two half sub-units are specularly connected at the M-line. Within each sub-sarcomer, the trajectory of titin span from the M to the Z-zone. The thick filaments are based on rigid myosin bound to three to six tinin molecules. The thin filaments are based on rigid actin bound tropomyosin and troponin and anchored to the Z-zone by the cross-linking protein α-actinin. Red: thin filaments: Green: thick filaments; Black: tinin; Yellow: alpha-actinin.

A novel approach to the description of the assembly mechanisms of complex, functional biostructures was recently presented (Ciferri and Crumbliss, 2018). The approach postulated the occurrence of a fundamental self-assembling mechanism, specific for any biostructure, associated with “engineered” strategies used by Nature to optimize functions. Using the latter approach, a new mechanism was suggested for the structure and the contraction of the sarcomer, the structural repeating unit of the myofibrils of striated muscles (Figure 1). The liquid crystallinity of mixtures of thick and thin filaments was regarded as the fundamental driving force for the structurition of single sarcomers.

Whereas, the mechanism of muscular contraction had been extensively investigated, much less is known about the assembly mechanism of the myofibril. In the present communication we extend our approach based on a fundamental mechanism and associated engineered modifications to the assembly of myofibrils, known to reach macroscopic dimensions. Published results on the self-assembly of neuronal axons evidenced that their fundamental self-assembling mechanism is the isodesmic supramolecular polymerization (Ciferri, 2016b). It will be shown here that the latter mechanism also drives the assembly of the myofibrils.

It is essential to appreciate the full relevance of the various self-assembly mechanisms postulated to drive the formation of elongated biostructures. These self-assembling mechanisms are intended to reflect specific *matter properties*. For instance, the liquid crystalline mechanism that controls the assembly of single sarcomers reflects the excluded volume of rigid particles, which necessarily induces one- or two-dimensional ordering in concentrated solutions (*shape recognition*, Flory, 1984; Ciferri, 2005). The isodesmic polymerization mechanism is instead based on the linear association of ditopic particles by soft attractive interactions (*chemical recognition*, Lehn, 2002; De Greef et al., 2009). Formal molecular elaborations of these assembling mechanisms were elaborated in the late 1900s. Nevertheless, matter properties and the above recognition patterns had to be operative even during the evolutionary stages.

## Isodesmic Supramolecular Polymerization

The multi-stage-open-association (MSOA) theory describes the growth of linear polymers that, following an irreversible propagation step, reach an equilibrium state at which the backward rand the forward reaction rates are in balance (Lahiri et al., 2015). No cooperative effects are involved in the *isodesmic* polymerization of consecutive monomers. In *molecular* polycondensation, which may be carried out under equilibrium or non-equilibrium conditions, a byproduct is observed, the association constant is usually large, and a large degree of polymerizations (DP) is obtained. In isodesmic s*upramolecular* polymerization, self-assembly without byproducts prevails, association constants are small (e.g., H-bonds), and large DPs would not ordinarily be expected. Nevertheless, resort to multiple supramolecular interactions has allowed the obtainment of DPs even larger than those obtained with molecular polycondensation (Ciferri, 2002; De Greef et al., 2009).

Theoretical elaborations of molecular and supramolecular polycondensation have accounted for their subtle differences (Zhao and Moore, 2003; van der Schoot, 2005). The theory of supramolecular polymerization yields the following approximate relationship (valid in the bulk phase) between the degree of supramolecular polymerization and the supramolecular binding constant K:

(1) $$\begin{array}{r} \text{DP}\sim {\text{K}}^{1/2} \end{array}$$

The association constant K appearing in Equation (1) represents the total binding interaction linking two neighboring monomers, irrespective of the number of supramolecular bonds linking each monomeric pair. When multiple interaction between neighboring pairs occurs, K is the product of the corresponding binding constants, reflecting the additivity of single binding free energies.

It is essential to appreciate the full relevance of Equation (1) that simultaneously express the strength of the supramolecular bond and the polymerization degree. The concept that multivalent interaction increases the *strength* of a pair-wise association is often encountered in the biochemical literature. For example, Krishnamurthy et al. (2006) have discussed the difference between “affinity “and an “avidity” constant, intended to respectively, represent the occurrence of single or multiple H-bonds interactions between two mono-functional molecules. In the present contest of bi-functional monomers polymerization, the association constant K in Equation (1) includes the “avidity” concept, but also allows the prediction of the length of the assembly.

Experimental results for a variety of supramolecular synthetic and biological model polymers have amply confirmed the above expectations regarding the conditions under which extremely large DP can be attained. Hilger and Stadler (1991) have reported the case of a polybutadiene polymer carrying phenylbutazone units additionally substituted with a carboxyl group. Only supramolecular oligomers with DP up to 20 were obtained. An independently assessed equilibrium constant, K ~ 500/M, showed fair correspondence with Equation (1). The use of multiple H-bonds was later shown by Meijer and coworkers to be the winning strategy for producing supramolecular polymers with large DP (De Greef et al., 2009). They terminated a linear polydimethylsiloxane polymer with ureidopyrimidone units, able to dimerize and stabilize a supramolecular polymer with main chain linkages based on four hydrogen bonds. The equilibrium constant derived from Equation (1) was in satisfactory agreement with the K value evaluated for the particular sequence of donor-acceptor H-bonds for dimerized ureidopyrimidone.

Non-isodesmic supramolecular polymerization involving intra or intermolecular cooperative effects are also known, and have been reviewed (Ciferri, 2002, 2005).

## Extension of MSOA to Biological Systems

### Axon

**Figure 2:**
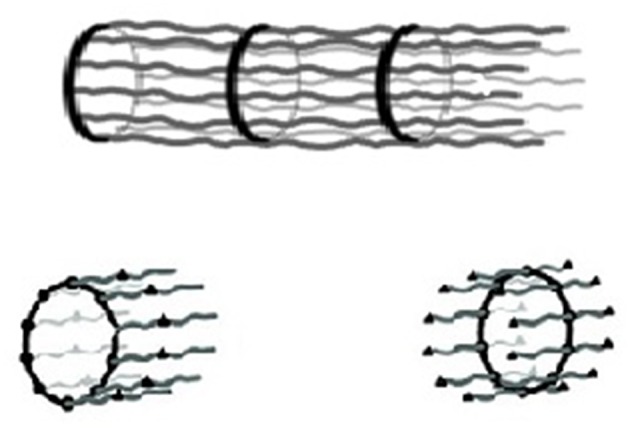
(Top) Schematic representation of the structure of the axon: a rigid sequence of adducin-capped actin rings connected by rigid spectrin tetramers with a periodicity along the axon axis of 180–190 nm. (Bottom) Possible monomeric units (Xu et al., 2013; Ciferri, 2016b).

The application of the isodesmic MSOA model to macroscopic biological structures was considered in the case of the neuronal axon (Ciferri, 2016a,b). Results are briefly summarized. The molecular structure, elucidated by high resolution fluorescence imaging techniques, evidenced a tubular organization based on a sequence of adducing-capped actin rings connected by a number (up to 10) of spectrin *tetramers* (Xu et al., 2013; Figure 2). The tetramers resulted from non-covalent association of alpha and beta spectrin *dimers* through their C and N terminals (Hill et al., 2014). The reported association constants for the spectrin dimer–dimer association is on the order of 10^4^/M (Brenmer and Korn, 1980), somewhat smaller than the constant for the actin -spectrin association. Therefore, the repeating unit of the supramolecular chain was identified as an actin ring decorated on both surfaces by spectrin dimers, polymerizing by the formation of tetramers (Figure 2).

In terms of Equation (1), the above constant for the formation of a single tetramer would only allow a DP in the order of 10^2^. However, a DP up to 10^7^ was suggested by the comparison of the macroscopic length of a full-grown axon with the nanometric length of a spectrin tetramer. Indeed, Equation (1) would predict a DP in the order of 10^7^ for the combined formation of about eight hydrogen bonds, each contributing a constant K ca. 10^4^/M. This result is consistent with the large number of spectrin tetramers that stabilize the axon structure in Figure 2.

### Myofibril

We analyze the possibility that the myofibril attains macroscopic dimensions by virtue of multiple supramolecular interactions between neighboring sarcomers. Details affecting the repeating sarcomeric unit and its binding constant are much more complex than in the case of the axon reviewed above. In fact, in addition to actin and a spectrin-type compound (α-actinin), other components occur in the sarcomer, notably titin, and myosin. Moreover, each sarcomer includes two half sub-units, specularly connected at the M-line (Figure 1).

Titin is a giant polymer (over 1 μ long) that spans the entire length of each sarcomer between the terminal Z-lines. Its tertiary structure includes 244 partially ordered domains separated by unstructured segments. Its sequence, including the gene sequence and the structuration of the domains, are adequately known (Bang et al., 2001; Krüger and Linke, 2011). As suggested by various investigators (Liversage et al., 2000), six titin molecules, firmly wound to each thick myosin filament in the central part of the sarcomer, terminate in the Z-zone.

α-actinin is primarily involved in the attachment of actin filaments to the Z-zone, but can establish direct bonds also with titin. The structure of α-actinin is based on a rod-like dimer of four antiparallel rigid spectrin-like domains having actin binding sites at each end of the dimer (Figure 1; Young et al., 1998; Sjöblom et al., 2008; Grison et al., 2017). Close to the actin binding domains, two pairs of cal-moduline type sites can be chemically activated to an open conformation that binds to the Z-repeat domain of titin. The actual bond between titin and α-actinin is localized in a 45-amino acid sequence located in the N-terminal region of titin (Z-repeat) and in the C-terminal region of α-actinin (Joseph et al., 2001).

Several authors have investigated the interaction between actin, titin and α-actinin. Grison et al. (2017) used dual-beam optical tweezers to study the mechanical behavior of a model compound that included calmodulin-like domain of α-actinin bound to the Z-repeats of titin. At the single-molecule level, the association of α-actinin and titin was found to be rater weak under the applied force. The decomposition constant K_d_, extrapolated at zero force, was found to be 4 ± 2 μM, comparable with other literature data suggesting K_d_ = 0.1–0.25 μM (Joseph et al., 2001). The corresponding binding constant K can be easily evaluated and is in the order of 10^4^/M, a value comparable to that reported above for the formation of the spectrin tetramer in the axon.

Supramolecular bonds between α-actinin and titin may in principle be established within the Z-zone of a given sarcomer, or between two neighboring sarcomers. The applicability of the supramolecular polymerization model requires the continuity of titin, the only polymer that spun the entire length of each sarcomer, along the myofibrillar axis. This continuity would require α-actinin bridges between titin residues of two neighboring sarcomers.

We can estimate the possible number of titin-titin bridges between two adjacent sarcomers by considerations based on the average lengths of the sarcomer and the myofibril. Assuming lengths of up to ca. 0.3 cm for a myofibril, and a maximum of ca. 3.0 μ for each sarcomer (Bertz et al., 2009), a DP in the order of 10^6^ is predicted. Using Equation (1) and the value K = 10^4^/M reported above for a single site interaction, we conclude that between two or three interacting sites must be involved to produce the predicted DP.

We explored the literature for data consistent with the above expectation. Using elaborate electron scanning microcopy, Liversage et al. (2000) compared the symmetry parameters of the thick and the thin filaments with the corresponding ratio of the of the number of these filaments. They concluded that only four terminals of the six titin molecules emerging from each thick filament would react with the thin filaments in the Z-zone. The remaining two were postulated *to* enter the adjacent sarcomer, supporting the conclusion based on Equation (1). Liversage might have been the first to discover the polymerization mechanism, but suggested instead a binding of the emerging titin terminals to the actin filaments in in neighboring sarcomers.

**Figure 3:**
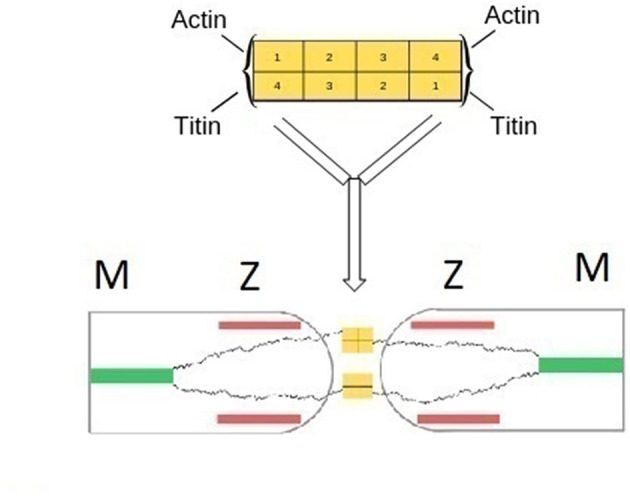
(Top) Schematic representation of the structure of α-actinin based on a rod-like dimer of four antiparallel rigid spectin-like domains. Binding of actin or tinin filaments occur at each end of the dimer (Grison et al., 2017). (Bottom) Schematic representation of the fusion of two Z-zones of neighboring half sarcomers and the formation of main-chain titin bonds. α-actinin bridges titin terminals of two adjacent sarcomers. Bridges between titin and actin filaments are omitted for clarity.

Assuming the above situation to prevail at both terminals, a bifunctional *symmetric* sarcomer would be formed having two tinin binding sites at each of its Z-terminals. The actual polymerization would occur by coupling with bifunctional α-actinin. The sequence involving the titin N terminals of adjacent sarcomers bridged by α-actinin, would follow the classical polymerization scheme of symmetric monomers … AA-BB-AA… The Z-zones of two adjacent sarcomers would actually merge, incorporating the two supramolecular bonds. A schematization is presented in Figure 3.

The substantial concordance between data obtained from the application of Equation (1), the actual sizes of the myofibril and the sarcomer, and the symmetry analysis by Liversage et al. (2000). support the plausibility of the MSOA model as the fundamental growth mechanism of the microfibril. The anchoring of titin to the Z and M zones and the thick filaments does not conflict with the MSOA mechanism. In supramolecular polymerization only the association of the terminal bonds matters, the size and the structuration of the repeating units are irrelevant. Indeed, the formation of the myofibril might be described as a supramolecular polymerization of a titin polymer “decorated” by the various components of the sarcomer.

## Engineered Strategies

While the elucidation of the fundamental polymerization mechanism of the myofibril was a relatively simple task, proper analysis of the molecular structure of the myofibril should include ta characterization of the engineered strategies used by nature to enhance muscle's functions. These engineered strategies primarily include bonds that anchor titin, the thin and the tick filaments to the sarcomer. Biochemical analysis has focused on the characterization the bonds between titin, the thin filaments and α-actinin in the Z-zone (Figure 3). The winding of titin around the thick segment was also characterized. Remarkably, the specific sequences involved in these interactions were often identified (Wang et al., 2018). Relatively less investigated has been the anchoring of titin and the thick filaments at the M-line. Proteins such as myomesin and M-protein were found to anchor titin and the thick filaments to the M-line, and to bridge the titin COOH terminals of the two half sarcomers (Wang et al., 2005). Complexes between titin, M-protein and nyosin have been identified by Martinfavli et al. (2017).

The sequence of the formation of the various components during fibrillogenesis was also investigated. Studies revealed that Z-bodies formed during the pre-myobrillar stage were remodeled during the following nascent and mature stages (Wang et al., 2005). α-actinin was present at all development stages and able to bind to the multiplicity of proteins (up to 25) that appeared during the mature stage. Teletonin (Bertz et al., 2009) appeared only at the mature stage. Tinin, instead, appeared at the nascent stage, which is consistent with its postulated role in the polymerization of the sarcomers.

The above chemical details of the engineered strategies do not, however, elucidate the “rationale” (or the know-why) of the anchoring interactions that evolved from the evolutionary stages. In our previous communication (Ciferri and Crumbliss, 2018), we suggested that the rationale for the multiple anchoring interactions occurring within each sarcomer should be assessed from consideration of the intended mechanical functions of the striated muscle. We point out the following examples. Anchoring of the thick and the thin filaments to the M and Z surfaces is an absolute requirement to produce a reversible and a reproducible unidimensional contraction along the fiber axis. Moreover, the rationale for the winding of titin along the thick filament is a strategy nature used to bypass the shape incompatibility of a flexible polymer within the ordered phase of a rigid one (Flory, 1984). Furthermore, the rationale for occurrence of the M-line is to allow the binding of two COOH terminals of two titin segments, making thus possible the formation of a symmetric repeating unit of the AA type. Detailed knowledge of the relevant binding sequences would afford only a know-how type information, useful but insufficient to offer a rationale for the engineered strategies.

The foregoing approach finds support in the Complexity Theory (Mitchell, 2011). The latter analyzes the behavior of complex systems when a unifying principle is not detectable from analysis of single components. In the case of the sarcomer, the unifying principle was assumed to be the requirement that the engineered features need to be consistent with the mechanical function of striated muscles. This principle has allowed a top-down approach to the interpretation of the anchoring modes of the main sarcomeric components.

## Conclusions

The assembly of the myofibril is well-described by the fundamental MSOA theory that relates the extent of grow to a supramolecular association constant.

The complex structure of the sarcomere, resulting from the superimposition of engineered features, is rationalized in terms of the intended mechanical properties of the striated muscle.

Our approach is at variance with conventional biochemical analysis and offers a perspective that might be expanded by future investigations.

## Data Availability

All datasets generated for this study are included in the manuscript and/or the supplementary files.

## Author Contributions

AC originated the approach based on the supramolecular polymerization. ALC offered substantial contribution inspired by the complexity theory.

## Contributions to the Field Statement

The identification of the strategies followed by Nature for the construction of some functional structures is the main contribution of this work. These strategies were analyzed in terms of fundamental assembling mechanisms, which reflect matter properties, and engineered modifications that optimize functions. In the case of the myofibril, the fundamental assembly mechanism was identified as the supramolecular polymerization of the sarcomer, regarded as the monomeric repeating unit of the striated muscle structure. The engineered features were associated to the mutual anchoring of the main sarcomeric components: actin, myosin, tinin, alpha-actinin. The rationale for the engineered features may be appreciated in terms of the intended mechanical properties of the system, rather than by conventional biochemical analysis. The perspective character of the contribution advocates the extension of the approach to other natural or man-made functional structures.

### Conflict of Interest Statement

The authors declare that the research was conducted in the absence of any commercial or financial relationships that could be construed as a potential conflict of interest.
